# Lower Amounts of Daily and Prolonged Sitting Do Not Lower Free-Living Continuously Monitored Glucose Concentrations in Overweight and Obese Adults: A Randomised Crossover Study

**DOI:** 10.3390/nu14030605

**Published:** 2022-01-30

**Authors:** Daniel P. Bailey, Charlotte A. Stringer, Benjamin D. Maylor, Julia K. Zakrzewski-Fruer

**Affiliations:** 1 Centre for Physical Activity, Health and Disease, Brunel University London, Uxbridge UB8 3PH, UK; 2 Division of Sport, Health and Exercise Sciences, Department of Life Sciences, Brunel University London, Uxbridge UB8 3PH, UK; 3 Institute for Sport and Physical Activity Research, School of Sport Science and Physical Activity, University of Bedfordshire, Bedford MK41 9EA, UK; charlotte.stringer1990@gmail.com (C.A.S.); bm259@leicester.ac.uk (B.D.M.); julia.fruer@beds.ac.uk (J.K.Z.-F.); 4 Diabetes Research Centre, University of Leicester, Leicester General Hospital, Leicester LE5 4PW, UK

**Keywords:** sitting, sedentary behaviour, physical activity, activity breaks, glycaemia, glucose

## Abstract

This study compared the short-term continuously monitored glucose responses between higher and lower amounts of prolonged sitting in overweight and obese adults under free-living conditions. In a randomised crossover design, 12 participants (age 48 ± 10 years, body mass index 33.3 ± 5.5 kg/m^2^) completed two four-day experimental regimens while wearing a continuous glucose monitor, as follows: (1) uninterrupted sitting (participants were instructed to sit for ≥10 h/day and accrue ≥7, 1 h sitting bouts each day), and (2) interrupted sitting (participants were instructed to interrupt sitting every 30 min during ten of their waking hours with 6–10 min of activity accrued in each hour). Linear mixed models compared outcomes between regimens. None of the continuously monitored glucose variables differed between regimens, e.g., 24 h net incremental area under the glucose curve was 5.9 [95% CI: −1.4, 13.1] and 5.6 [95% CI: −1.7, 12.8] mmol/L∙24 h, respectively (*p* = 0.47). Daily sitting (−58 min/day, *p* = 0.001) and sitting bouts lasting ≥30 min (−99 min/day, *p* < 0.001) were significantly lower and stepping time significantly higher (+40 min/day, *p* < 0.001) in the interrupted sitting than the uninterrupted sitting regimen. In conclusion, lower amounts of daily and prolonged sitting did not improve free-living continuously measured glucose among overweight and obese adults.

## 1. Introduction

Overweight and obesity contribute significantly to insulin resistance and glucose intolerance [1,2]. Thus, interventions to aid with the management of glucose metabolism in these individuals is important to reduce the risk of insulin resistance and subsequent cardiometabolic disease [1,3]. Indeed, repeated elevations in glucose concentrations can lead to a plethora of metabolic disturbances that increase the risk of type 2 diabetes mellitus (T2DM) and cardiovascular disease, including insulin resistance, pancreatic β-cell deficiency, oxidative stress and endothelial dysfunction [4,5]. This is important because it is estimated that 463 million adults worldwide have T2DM [6]. Cardiovascular disease is estimated to cause 17.9 million deaths per year, making it the leading global cause of mortality [7].

High volumes of sedentary behaviour are associated with increased risk of T2DM and cardiovascular disease, which may be independent of moderate-to-vigorous physical activity [8,9]. This increased risk may be via the detrimental associations that higher sedentary time has with cardiometabolic disease risk markers, such as glucose tolerance and insulin resistance [10,11]. The manner in which sedentary time is accumulated also has implications for cardiometabolic health. A single day of prolonged sitting led to a 39% reduction in insulin action compared with an active day of standing and ~10,000 steps in healthy adults [12]. An increased frequency of interruptions to sitting is also associated with improved fasting glucose and glucose tolerance [11]. Interrupting sitting in controlled laboratory environments with 2 to 5 min of standing, light-intensity walking or moderate-intensity walking every 20 to 30 min attenuates postprandial glucose responses over a single day in individuals who are healthy, overweight/obese and/or have impaired glucose tolerance [13,14,15,16]. Dunstan et al. [15] reported that overweight or obese individuals with normal fasting glucose had similar reductions (24–30%) in postprandial glucose in response to interrupting sitting for 2 min every 20 min with light or moderate-intensity walking. It appears that the effects of interrupting sitting on glucose metabolism may be more pronounced in participants with adverse cardiometabolic health profiles and in individuals who are overweight/obese with impaired or apparently normal glucose levels [13,15,17]. Reducing prolonged sitting in individuals who are overweight and obese may thus be important for optimal cardiometabolic health.

The ability to achieve improvements in glucose via reductions in prolonged sitting, outside of laboratory settings, is not well understood. Continuous glucose monitoring offers a unique opportunity to examine glucose responses in free-living settings that may be caused by reducing and breaking up sitting time. Replacing 4.7 h/day of sitting in an imposed sedentary regimen with 2.5 h and 2.2 h of standing and light-intensity walking in a “sit less” regimen over four days resulted in a significant 36% lower 24 h glucose concentration in participants with T2DM [18]. Another study in participants with T2DM did not see any significant reduction in 24 h glucose concentrations when sitting was interrupted with four bouts of walking within the hour immediately after breakfast, lunch and dinner, compared to a day of normal habitual activity [19]. Yet, differences in sitting and activity between the conditions may have been insufficient to detect significant effects on glucose; indeed, participants interrupted sitting only in the hour after each meal and the control condition was habitual activity as opposed to an imposed increase in sitting in the study by Duvivier et al. [18]. In overweight adults with normal fasting glucose concentrations, four days of sitting for 7.6 h, standing for 4.0 h and light-intensity walking for 4.3 h per day led to significant improvements in insulin sensitivity and reductions in insulin during an oral glucose tolerance test compared with four days of sitting for 13.5 h, standing for 1.4 h and light-intensity walking for 0.7 h per day [20]. The effects of interrupting sitting on free-living continuous glucose concentrations, however, has not been evaluated in overweight and obese participants. This gap in research should be addressed to extend knowledge with regard to reducing and breaking up sitting being used as a potential intervention strategy for reducing the risk of T2DM and cardiovascular disease in this ‘at risk’ population [21].

The primary aim of this study, therefore, was to compare continuously monitored glucose responses between higher and lower amounts of daily and prolonged sitting under free-living conditions among overweight and obese individuals. The secondary aim was to evaluate sitting, standing and stepping responses to the free-living experimental protocols.

## 2. Materials and Methods

### 2.1. Study Design and Overview

A within-subjects randomised crossover design was employed and reported following Consolidated Standards of Reporting Trials guidance [22]; see checklist in Supplementary Material Table S1. Following preliminary tests, participants took part in two, 4-day long activity regimens in free-living conditions, as follows: (1) uninterrupted sitting and (2) interrupted sitting. Participants were randomised to regimen order by the research team using a simple randomisation method via an online tool (www.randomizer.org) (accessed on 1 November 2016). There was a 72 h washout period between regimens to avoid potential insulin sensitivity carryover effects that have been observed ≥48 h after a single exercise session [23]. The study was reviewed and approved by the University of Bedfordshire Institute for Sport and Physical Activity Research Ethics Committee (no. 2017ISPAR004) and conformed to the Declaration of Helsinki. Participants provided written informed consent prior to any testing procedures.

### 2.2. Participants

Participants were 23–63 years old with overweight or obesity as defined by a body mass index (BMI) of 25 to 45 kg/m^2^ [24] and a waist circumference of ≥102 cm for men and ≥88 cm for women [25]. Habitual sitting time needed to be at least 7 h/day and moderate-to-vigorous physical activity less than 150 min/week to be included in the study; this was screened using a domain specific sitting time questionnaire [26] and the International Physical Activity Questionnaire [27], respectively; these criteria were used in anticipation that metabolic responses to manipulations in sitting would be greatest in individuals who sat more and were physically inactive [20,28]. Exclusion criteria were working night shifts, current or recent smoker, contraindications to physical activity, fitted with an artificial pacemaker, diabetes, a known blood borne disease, recreational drug use, alcohol addiction, pregnancy, or taking glucose or lipid-lowering medication. Participants were recruited at the University of Bedfordshire and from the local community using adverts and word-of-mouth.

### 2.3. Sample Size

The primary outcome for this study was 24 h glucose net incremental area under the curve. To detect an effect size of d = 1.27 between conditions, at least 10 participants were required to achieve 90% power based on a two-tailed alpha of 0.05 and a within-person correlation of 0.5. The effect size for the calculations was informed by previous experimental research [13,15,18].

### 2.4. Preliminary Visit

A researcher met with participants at their workplace, a community location, or at the University of Bedfordshire Sport and Exercise Laboratories for preliminary measures. This always occurred on a Monday, 24 h before the commencement of the first experimental regimen. Height was measured to the nearest 0.1 cm and body mass to the nearest 0.1 kg. Bioelectrical impedance analysis was used to provide a valid estimate of body fat% (Tanita BC-418 Segmental Body Composition Analyzer; Tanita Corp., Tokyo, Japan) [29]. The midpoint between the lowest rib and the iliac crest was located to take a measure of waist circumference to the nearest 0.1 cm. A FreeStyle Libre (Abbott Diabetes Care, Ltd., Witney, UK) flash glucose monitor (FGM) was inserted at the midline of the upper arm for each participant and an activPAL3 activity monitor (PAL Technologies, Glasgow, UK) was attached to the thigh. Following this, participants were informed of the order of their experimental regimens and provided verbal and written guidance on how to complete the protocols.

### 2.5. Experimental Design

**Figure 1:**
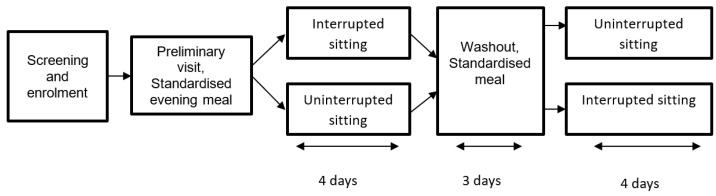
Schematic of study protocol.

Figure 1 shows the experimental protocol. The first experimental regimen took place Tuesday to Friday. There was then a 72 h washout period and the second experimental regimen took place the following Tuesday to Friday. An activity log was provided so participants could record the amount of sitting, standing or physical activity each hour throughout each of the experimental regimens. This was provided in an attempt to help participants visualise their behaviour and thus encourage compliance with the protocols. The two, 4-day regimens were based on previous experimental research in which reducing and breaking up sitting led to acute improvements in cardiometabolic health under free-living conditions [20,30]. They were as follows:

#### 2.5.1. Uninterrupted Sitting Regimen

During this regimen, participants were instructed to (1) increase sitting as much as possible, (2) sit for ≥10 h/day, and (3) engage in no more than a combined total of 1.5 h/day of standing and stepping. Participants were asked to sit continuously without any breaks for 7 of the 10 h in which they were asked to remain seated (i.e., they were required to accumulate 7 bouts of uninterrupted sitting that were each ≥1 h in duration), except for visiting the toilet. They were free to select when in the day they engaged in these 7 h of uninterrupted sitting. This was to encourage the accumulation of sitting in prolonged bouts. During the other 3 h, participants were instructed to interrupt their sitting no more than once per hour for a maximum of 15 min at a time. This allowed time to engage in activities of daily living, such as getting dressed, bathing, cleaning and cooking. They were also asked to travel by car or public transport wherever possible to limit activity accumulated when travelling.

#### 2.5.2. Interrupted Sitting Regimen

For the interrupted sitting regimen, participants were asked to interrupt sitting bouts at least once every 30 min with standing or physical activity for a duration of 3–5 min each time. They were asked to interrupt sitting in at least 10 of their waking hours (i.e., at least 20 activity breaks per day) and accumulate a total of 6–10 min of interruptions in sitting in each of these hours. This gave participants flexibility as to which hours they interrupted their sitting during the day (e.g., a mix of at work and during leisure time). The frequency and duration of interruptions to sitting was advised based on previous controlled laboratory and free-living studies that observed improvements in postprandial glucose with similar protocols [13,17,18]. Participants were also instructed to engage in ≥1.5 h of standing and/or physical activity across each day. A range of smartphone and computer apps were suggested to be used for alerts/reminders to interrupt sitting at least every 30 min. Demonstrations and written suggestions for activities to engage in when interrupting sitting were provided. This included standing, walking, simple resistance activities (e.g., knee lifts, half squats, calf raises, lunges), stair climbing and repeated sit-to-stand transitions). Breaking up sitting with these activities has resulted in significantly attenuated postprandial glucose concentrations over a single day in laboratory conditions [13,14,17]. To improve compliance and ecological validity, participants could perform an activity that they felt was best suited to the situation or environment they were in at the time.

#### 2.5.3. Standardisation of Dietary Intake and Physical Activity

Participants were asked to avoid engaging in moderate-to-vigorous physical activity and from consuming alcohol for 48 h before their first experimental regimen and throughout the rest of the experimental protocol. A standardised instant pasta meal (464.0 ± 2.0 kcal, carbohydrate 80.7 ± 2.8 g, protein 18.2 ± 1.2 g, and 7.0 ± 1.1 g fat) was provided for participants to consume at the same time in the evening before each 4-day regimen (Figure 1). Participants used electronic scales (Salter Disc Electronic Kitchen Scale, HoMedics Group Ltd., Tonbridge, UK) to weigh all food and drink intake throughout the first 4-day regimen they took part in. They recorded the time and volume of dietary intake in a food diary and were asked to replicate this dietary intake exactly during the second 4-day regimen. Participants were instructed to consume at least three meals containing ≥50% of carbohydrate (examples of such meals were provided to each participant) on each experimental regimen day as well as any snacks they wanted to consume to encourage multiple glucose excursions throughout the day that were amenable to change via the experimental regimens. 

### 2.6. Measurements

#### 2.6.1. Sitting, Standing and Stepping

Sitting, standing, stepping and postural transitions were measured throughout the 11-day experimental period using the activPAL3 activity monitor, which provides valid measures for these outcomes [31,32]. The activPAL3 was worn on the anterior of the right thigh and was attached to the skin using an adhesive dressing (Hypafix Hypoallergenic Tape; BSN Medical Limited, Hull, UK). The device was waterproofed with a nitrile sleeve and a Hypafix dressing to enable continuous wear. To aid with processing of data from the activPAL, participants used a diary to record the time they woke up and got out of bed, when they were at work, went to bed and to sleep, and times that the device was removed [33]. Data was processed using an automated algorithm [34] executed within STATA (StataCorp LLC, College Station, TX, USA) to derive the following outcomes: sitting time, number and duration of short sitting bouts (0–30 min), number and duration of prolonged sitting bouts (≥30 min and ≥60 min), standing time, number of sit-upright transitions, stepping time, and number of steps. A valid wear day was accepted when wear time was >10 h, there were >500 steps recorded, and no more than 95% of the recorded data was in one activity category (i.e., sitting, standing or stepping) [34]. For inclusion in the analysis, at least one valid day of wear was required.

#### 2.6.2. Flash Glucose Monitoring

The FreeStyle Libre was used to measured flash glucose concentrations on a continuous basis throughout the study. During the preliminary visit, a Freestyle Libre sensor was inserted subcutaneously at the midline of the back of the upper arm in line with manufacturer guidelines. The FreeStyle Libre provides valid measures of interstitial glucose compared to reference capillary blood glucose values from the YSI analyzer (Yellow Springs Instrument, Yellow Springs, OH, USA) and is consistently accurate over 14 days [35]. Participants thus wore the FGM sensor continuously throughout the 11-day experimental period. The device samples and stores interstitial glucose concentrations every 15 min. The data was transferred to a Freestyle Libre reader and exported into Microsoft Excel at the end of the experimental protocol by a researcher. Data was processed using a custom R script. Days in which the device recorded data for <70% of the 24 h period were classified as invalid wear. A minimum of one valid day in each experimental regimen was required for inclusion in the analysis. The following glucose metrics were calculated across 24 h periods for each of the experimental regimens starting with each participant’s wake time on the first day of monitoring: (a) mean glucose concentrations, (b) total area under the curve (AUC) calculated using the trapezoidal method, (c) net incremental area under the curve (iAUC) calculated by subtracting the waking baseline glucose concentration for each day from total AUC, and (d) glycaemic variability, i.e., glucose coefficient of variation (CV). Mean glucose concentrations, glucose total AUC and glucose iAUC were also calculated for waking hours only. 

### 2.7. Statistical Analysis

All statistical analysis was completed using SPSS version 22 (IBM, Armonk, NY, USA). Q-Q plots were visually inspected to assess normality of the data prior to analysis. All variables were deemed to be normally distributed. The main effect of experimental regimen (uninterrupted sitting versus interrupted sitting), experimental regimen day, and the regimen x day interaction for the study outcomes were analysed using linear mixed models. Fixed factors in the model were experimental regimen, day, and covariates. Participant ID was initially entered as a random factor in each model, but this term was subsequently removed as this covariance parameter was redundant. For glucose AUC models, baseline glucose concentration (for each day) was entered as a covariate, while waking wear time was entered as a covariate in sitting, standing and stepping models. Unless stated otherwise, data is presented as mean (95% confidence interval [CI]). The alpha level for statistical significance was *p* ≤ 0.05. Effect sizes (Cohen’s d) were calculated to indicate the magnitude of difference for significant outcomes with d < 0.2, 0.2–0.49, 0.5–0.79 and ≥0.8 considered trivial, small, medium and large effects, respectively [36].

## 3. Results

### 3.1. Study Sample

**Figure 2:**
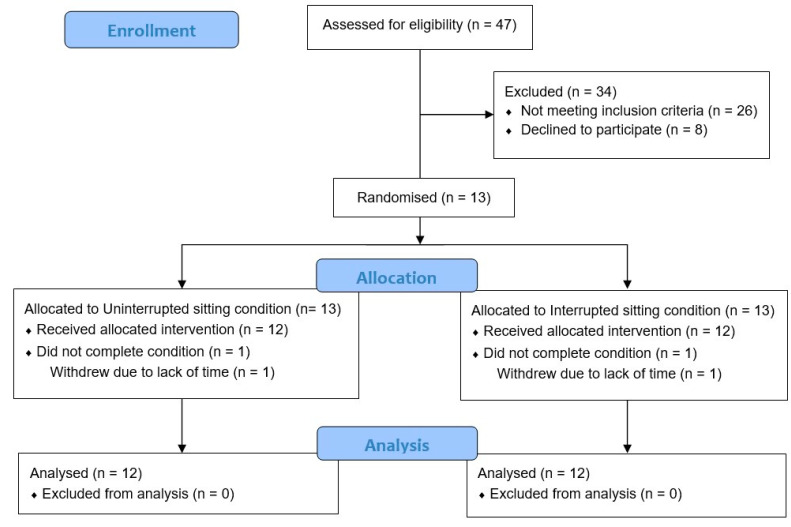
Participant flow throughout the study.

Recruitment of participants took place between November 2016 and April 2017. Participant flow throughout the study is shown in Figure 2. Following screening, there were 13 participants enrolled into the study. One participant withdrew prior to commencing the experimental protocol. Analysis was thus conducted for the 12 participants who completed the study; their descriptive characteristics can be seen in Table 1. 

### 3.2. Dietary Intake

The mean carbohydrate, fat and protein intake that participants self-reported in their food diary during the experimental regimens was 294 ± 109 g, 86 ± 38 g and 86 ± 25 g/day, respectively. Carbohydrate, fat and protein intake accounted for 63%, 19% and 18%, respectively, of total dietary intake. Total energy intake was 9460 ± 2832 kJ/day.

### 3.3. Sitting, Standing and Stepping

ActivPAL data was unavailable for one participant on days 3 and 4 of the interrupted sitting regimen. All other monitoring days were valid for all other participants across both regimens. As shown in Table 2, daily sitting time was significantly lower in the interrupted sitting regimen than in uninterrupted sitting (d = 0.65). Time spent in prolonged ≥30 min sitting bouts was also significantly lower in the interrupted sitting than the uninterrupted sitting regimen (d = 1.29), as was time spent in prolonged ≥ 60 min sitting bouts (d = 1.99). Participants accumulated significantly more time in short sitting bouts in the interrupted sitting regimen than in uninterrupted sitting (d = 0.82). Although the number of short sitting bouts and the number of ≥30 min prolonged sitting bouts did not differ significantly between regimens, the number of ≥60 min prolonged sitting bouts was significantly lower in the interrupted sitting than the uninterrupted sitting regimen (d = 2.38). The number of sit-upright transitions per day did not differ significantly between regimens. The main effects of day (all *p* > 0.17; data not shown) and the regimen x day interaction effects (all *p* > 0.37) were not significant for any of the sitting or sit-upright transition variables (see Supplementary Material Table S2).

Standing time was not significantly different between the uninterrupted sitting and interrupted sitting regimens. In the interrupted sitting regimen, participants spent significantly more time stepping than during the uninterrupted sitting regimen (d = 2.49). Correspondingly, the number of steps was also significantly higher in the interrupted sitting than the uninterrupted sitting regimen (d = 3.06). The main effects of day (all *p* > 0.32; data not shown) and the regimen x day interaction effects (all *p* > 0.32) were not significant for standing time, stepping time, or number of steps (see Supplementary Material Table S2).

### 3.4. Flash Glucose Monitoring

The FGM monitor was active for an average of 97.1% of the wear period during the study. There were three missing days of data for one participant and one missing day for three participants; data from all participants therefore met the criteria for inclusion in the analysis. There was no significant difference in 24 h mean glucose concentrations, glucose total AUC, glucose iAUC or CV between the uninterrupted sitting and interrupted sitting regimens (see Table 3). There was no main effect of day for 24 h mean glucose concentrations, total AUC or iAUC (*p* = 0.65, 0.59 and 0.94, respectively; data not shown). There was a main effect of day for glucose CV (*p* = 0.02), which was significantly lower by 3.0% on day 4 than day 3 (*p* = 0.04; data not shown). There were no significant experimental regimen x day interaction effects for 24 h mean glucose concentrations, total AUC, iAUC or CV (*p* = 0.47, 0.71, 0.73 and 0.84, respectively; see Supplementary Material Table S3).

During waking hours, there was no main effect of experimental regimen for mean glucose concentrations, glucose total AUC, glucose iAUC or CV; see Table 3. The main effect of day was not significant during waking hours for mean glucose concentrations, glucose total AUC, or iAUC (*p* = 0.56, 0.72 and 0.95; data not shown). There was a main effect of day for glucose CV (*p* = 0.01), which was significantly lower by 3.9% on day 4 than day 1 (*p* < 0.01; data not shown). The regimen x day interaction effect was not significant for waking hours mean glucose concentrations, total AUC, iAUC or CV (*p* = 0.95, 0.79, 0.84 and 0.98, respectively; see Supplementary Material Table S3).

## 4. Discussion

The main finding in this study was that, in overweight and obese individuals, lower amounts of daily sitting and prolonged sitting did not improve continuously monitored glucose concentrations over four days when compared with prolonged sitting when self-implemented under free-living conditions. Based on this finding, public health recommendations to reduce and interrupt sitting may not be effective in the short term for improving continuous glucose. These findings are in contrast to a similar study in individuals with T2DM who experienced a 36% reduction in 24 h glucose iAUC when 4.7 h of sitting was replaced with standing and light-intensity walking over four days [18]. The discrepancies in our findings when compared with this previous research may be because there were more pronounced differences in sitting and activity between the two regimens in the study by Duvivier et al. [18]. Specifically, sitting was 58 min/day lower and stepping 40 min/day higher in the interrupted sitting than the uninterrupted sitting regimen, with no difference in standing. In the Duvivier et al. [18] study, sitting was 282 min/day lower and stepping and standing higher by 132 min/day and 150 min/day, respectively, in the “sit less” than the “sitting” regimen. In another study that compared a control condition of normal habitual activity to interrupting sitting with walking every 30 min for the two hours after breakfast, lunch and dinner, free-living 24 h glucose concentrations were unaffected in participants with T2DM [19]. This may have been because participants did not significantly reduce their daily sitting time, although stepping time was significantly higher by 25 min/day in the condition of breaking up sitting [19]. In the context of these findings, a greater reduction in sitting than the 58 min/day reported in the present study may be required to achieve improvements in continuously monitored 24 h glucose concentrations.

The present study found that lower amounts of daily sitting and prolonged sitting did not affect continuously monitored glucose levels during waking hours, when participants would have been predominantly in a postprandial state. In contrast to this, Blankenship et al. [19] reported significant reductions in total AUC for glucose and a trend for lower mean glucose during waking hours in response to interrupting sitting compared with normal habitual activity. This is despite larger reductions in daily sitting and a higher duration of stepping time in the current study. It is thus plausible that participants living with T2DM may be more sensitive than overweight and obese participants with normal glucose control in terms of beneficial postprandial (i.e., waking hours) glucose responses to reducing and interrupting sitting. The findings of the present study should also be considered in the context of the metabolically healthy obese phenomenon, which suggests that not all overweight and obese individuals are metabolically impaired [37]. Greater reductions in sitting and increases in physical activity may thus be needed in this type of participant for free-living glucose benefits. 

Participants in the present study significantly reduced the time they spent in prolonged ≥30 and ≥60 min sitting bouts by 99 min/day and 63 min/day, respectively. Prolonged sitting time in the study by Blankenship et al. [19] was not significantly reduced in their breaks condition, while it was not reported in the study by Duvivier et al. [18]. Based on these findings, it appears that reducing prolonged sitting through activity interruptions may not impart beneficial effects on continuously monitored glucose under free-living conditions. Glucose benefits may thus only be realised if the number of sitting interruptions is increased, or as suggested earlier, participants are at the lower end of the metabolic health spectrum. The present study, however, suggests that it was challenging for participants to change the number of interruptions (i.e., sit-upright transitions) in sitting as these were similar between regimens. This could be due to competing tasks at work or home, for example, that may dictate their sitting and activity-related behaviours. It is not possible to make comparisons to the study by Duvivier et al. [18] in this regard, as the number of sitting interruptions was not reported. Similar findings were seen in the study by Blankenship et al. [19] in which the number of interruptions in sitting during the breaks condition did not differ compared with a habitual activity condition. As research in this field is in its infancy, further studies that explore participants’ ability to manipulate their sitting behaviour in free living conditions and the effects that this has on glucose responses are needed to appropriately inform public health and clinical care guidelines.

The reasons that reductions in total and prolonged sitting time did not improve glucose responses is not clear, especially as reducing prolonged sitting in laboratory-based studies has attenuated postprandial glucose responses in overweight and obese individuals [13,15]. Thus, the issue of compliance may be important when attempting to translate findings from controlled, laboratory settings into free-living settings. Additionally, the intensity of the physical activity breaks was not controlled in the present study, which may have heighted the variability in individual glucose responses to the regimens, limiting the ability to detect significant effects when compared with previous laboratory work. It could also be postulated that, as opposed to reducing time spent in prolonged sitting bouts, interrupting sitting more regularly and reducing the number of prolonged sitting bouts, which were not achieved in the present study, are required. This may help to maintain permeability of muscle cells to glucose via contraction-mediated pathways and upregulation of genes that are involved in carbohydrate metabolism and translocation of the glucose transporter protein GLUT-4 [38,39]. Furthermore, although continuous glucose monitoring provides the opportunity to evaluate glucose responses to interrupting sitting under free-living conditions, these devices do not provide any indication with regard to the effects that an intervention might have on insulin sensitivity, which may be more amenable to changes in sitting time. Indeed, substituting total sitting time and the number of prolonged sitting bouts with corresponding increases in standing and light-intensity walking over four days under free-living conditions significantly improved insulin sensitivity during an oral glucose tolerance test that took place the morning after the experimental regimen ended in overweight and obese participants [20]. The present investigation did not assess glucose tolerance or insulin resistance status of the sample. The participants may have been metabolically ‘healthy’ in the context of these measures [37], which may mean there is limited potential for improving glucose in response to interrupted sitting. Nonetheless, this study did demonstrate that it was possible to favourably manipulate overweight and obese participants’ sitting, prolonged sitting and stepping, which, if repeated, could improve metabolic health in the longer term [40]. Future research should therefore consider whether the reductions in daily and prolonged sitting could affect insulin responses under ‘free-living’ conditions and investigate the long-term effects of such interventions.

The main strengths of this study include the evaluation of continuously monitored glucose concentrations under free-living conditions in response to manipulations in daily and prolonged sitting using a randomised crossover design, which enhanced ecological validity when compared with controlled laboratory studies. Furthermore, sitting, standing and stepping were continuously monitored throughout the experimental protocol and dietary intake was standardised between the regimens, with energy intake data suggesting minimal under-reporting. Despite differences in daily and prolonged sitting time and stepping time, the number of sitting interruptions did not differ between the experimental regimens. It is thus not possible to determine the effects that interrupting sitting combined with reduced daily and prolonged sitting and increased stepping may have on continuously monitored glucose in this study. Further, it may be more feasible to ask participants to interrupt their sitting more often in relation to their normal habitual activity as opposed to asking them to limit their sitting interruptions in an imposed uninterrupted sitting regimen. As the participants in this study were sedentary and physically inactive, another limitation is the ability to generalize the findings to individuals who may sit less and meet physical activity guidelines.

## 5. Conclusions

The findings of this study suggest that although it is possible to manipulate sitting and stepping under free-living conditions, lower volumes of daily and prolonged sitting and increases in stepping do not improve continuous glucose concentrations over the short-term in overweight and obese, but otherwise healthy, individuals. Future interventions should explore the longer-term effects of reducing and interrupting sitting on glucose metabolism in this population group.

## Figures and Tables

**Table 1:** Participant characteristics (*n* = 12).

Variables	
Female (*n*)	67% (*n* = 8)
Age (years)	48 ± 10
Body weight	93.9 ± 13.8 kg
Body mass index (kg/m^2^)	33.3 ± 5.5
Body fat%	39.4 ± 8.2
Waist circumference (cm)	107.8 ± 9.3
Fasting glucose (mmol/L)	5.4 ± 0.9

Data presented as mean ± SD.

**Table 2:** Sitting, standing and stepping outcomes during the experimental regimens (*n* = 12).

	Uninterrupted Sitting	Interrupted Sitting	Main Effect of Condition (*p* Value)
Daily sitting time (min/day)	671.2 (584.9, 757.3)	613.5 (527.2, 699.8)	<0.01
Time in short 0–30 min sitting bouts (min/day)	284.8 (229.7, 340.0)	331.9 (276.5, 387.2)	0.01
Time in prolonged ≥ 30 min sitting bouts(min/day)	385.9 (311.9, 459.9)	286.6 (212.2, 360.8)	<0.01
Time in prolonged ≥ 60 min sitting bouts(min/day)	215.0 (156.7, 273.3)	93.9 (34.9, 152.9)	<0.01
Sit-upright transitions (*n*)	50.2 (45.0, 55.3)	52.1 (46.9, 57.3)	0.35
Short sitting bouts (*n*)	43.6 (37.7, 49.5)	46.1 (40.2, 52.0)	0.25
Prolonged ≥ 30 min sitting bouts (*n*)	6.8 (5.3, 8.2)	6.2 (4.8, 7.6)	0.13
Prolonged ≥ 60 min sitting bouts (*n*)	2.6 (1.9, 3.2)	1.0 (0.4, 1.7)	<0.01
Standing time (min/day)	236.0 (155.6, 316.5)	254.0 (173.5, 334.6)	0.21
Stepping time (min/day)	80.9 (65.5, 96.4)	120.8 (105.3, 136.3)	<0.01
Total steps (*n*)	6491 (5529, 8354)	10,987 (9562, 12,412)	<0.01

Data displayed as mean (95% confidence interval).

**Table 3:** Glycaemic responses during the experimental regimens (*n* = 12).

	Uninterrupted Sitting	Interrupted Sitting	Main Effect of Condition (*p* Value)
**24 h period**			
Mean glucose concentration (mmol/L)	5.6 (5.1, 6.1)	5.6 (5.1, 6.1)	0.42
Glucose total AUC (mmol/L∙24 h)	131.9 (125.1, 138.6)	134.8 (128.0, 141.6)	0.47
Glucose iAUC (mmol/L∙24 h)	5.9 (−1.4, 13.1)	5.6 (−1.7, 12.8)	0.85
Coefficient of variation, %	19.0 (16.7, 21.3)	18.8 (16.5, 21.1)	0.78
**Waking hours**			
Mean glucose concentration (mmol/L)	5.9 (5.4, 6.4))	5.8 (5.3, 6.3)	0.56
Glucose total AUC (mmol/L∙waking hours)	101.2 (93.1, 109.2)	100.2 (92.2, 108.3)	0.63
Glucose iAUC (mmol/L∙waking hours)	6.8 (1.2, 12.3)	5.6 (0.0, 11.1)	0.37
Coefficient of variation, %	19.4 (17.2, 21.6)	19.6 (17.3, 21.9)	0.79

Data displayed as mean (95% confidence interval). AUC, area under the curve; iAUC, net incremental area under the curve.

## Data Availability

The data presented in this study are available on request from the corresponding author.

## Supplementary Materials

The following supporting information can be downloaded at: https://www.mdpi.com/article/10.3390/nu14030605/s1, Table S1: CONSORT 2010 checklist of information to include when reporting a randomised trial; Table S2: Sitting, standing and stepping outcomes across the experimental regimen days (*n* = 12); Table S3: Glycaemic responses across the experimental regimen days (*n* = 12).
